# Supplementary material for: Chemokine CXCL10 Modulates the Tumor Microenvironment of Fibrosis-Associated Hepatocellular Carcinoma
Source: Int J Mol Sci. 2022 Jul 23;23(15):8112. doi: 10.3390/ijms23158112 (PMC9329882; doi:10.3390/ijms23158112)
Supplement: Supplementary file 1 [file ijms-23-08112-s001.zip › ijms-1764906-supplementary.pdf]

Table S1. Key resources table.

REAGENT or RESOURCE	SOURCE	IDENTIFIER
**Antibodies**		
Anti-Rabbit IgG (Heavy and Light Chains)- Alexa Fluor 488	eBioscience	#Cat A-11008, RRID:AB_143165
Anti-Rabbit IgG (Heavy and Light Chains)- Alexa Fluor 546	eBioscience	#Cat A-11010, RRID:AB_2534077
Anti-Rat IgG (Heavy and Light Chains)- Alexa Fluor 488	eBioscience	#Cat A-21208, RRID:AB_141709
Anti-Rat IgG (Heavy and Light Chains)- Alexa Fluor 546	eBioscience	#Cat A-11081, RRID:AB_141738
aSMA (TSA Dye Cy 3)	Abcam	#Cat ab32575 RRID:AB_722538
B220-APC-Cy7 (CD45R)	eBioscience	#Cat A15399, RRID:AB_2534413
Biotin anti-mouse Lineage Panel	Biolegend	#Cat 133307, RRID:AB_11124348
Biotinylated Goat anti Rabbit IgG Antibody	Vector Laboratories	#Cat BA-1000, RRID:AB_2313606
CD103-PE	BD Bioscience	#Cat 557495, RRID:AB_396732
CD115-APC	eBioscience	#Cat 17-1152-82, RRID:AB_1210789
CD11b-eFluor450	eBioscience	#Cat 48-0112-82, RRID:AB_1582236
CD11b-PE	eBioscience	#Cat 12-0112-82, RRID:AB_2734869
CD11c-PE-Cy7	eBioscience	#Cat 25-0114-82, RRID:AB_469590
CD25-Alexa Fluor 700	eBioscience	#Cat 56-0251-80, RRID:AB_891424
CD3-PE-Cy7	eBioscience	#Cat 25-0031-82, RRID:AB_469572
CD4 (TSA Dye Cy 3.5)	Abcam	#Cat ab196159
CD45-BV510	BD Bioscience	#Cat 563891, RRID:AB_2734134
CD4-FITC	eBioscience	#Cat 11-0041-85, RRID:AB_464893
CD62L-APC-Cy7	eBioscience	#Cat 47-0621-82, RRID:AB_1603256
CD8 (TSA Dye Cy 5)	Bioss	#Cat bs-0648R RRID:AB_10857537
CD86-Biotin	eBioscience	#Cat 13-0862-82, RRID:AB_466525
CD8a-PerCP-Cy5.5	eBioscience	#Cat 45-0081-82

Cleaved Caspase 3	Cell Signaling	#Cat 9661S, RRID:AB_2341188
Cleaved Caspase 3 (TSA Dye Cy FITC)	Abcam	#Cat ab49822 RRID:AB_868673
Collagen IV (TSA Dye Cy 5.5)	Novotec	#Cat 20451 RRID:AB_10576569
F4/80-APC	eBioscience	#Cat 17-4801-82, RRID:AB_2784648
F4/80-PE-Cy7	eBioscience	#Cat 25-4801-82, RRID:AB_2784648
FoxP3-APC	eBioscience	#Cat 17-5773-82, RRID:AB_469457
Goat anti Rabbit IgG HRP polyclonal Antibody	Dako	#Cat P0448, RRID:AB_2617138
Ki-67	Cell Signaling	#Cat 12202S, RRID:AB_2620142
Ki67 (TSA Dye Cy FITC)	Abcam	#Cat ab15580 RRID:AB_443209
Ly6C-PerCP-Cy5.5	BD Bioscience	#Cat 560525, RRID:AB_1727558
Ly6G-FITC	eBioscience	#Cat 11-9668-80, RRID:AB_2572531
MHCII-Alexa Fluor 700	eBioscience	#Cat 56-5321-82, RRID:AB_494009
NK1.1-PE	BD Bioscience	#Cat 553165, RRID:AB_394677
ß-Actin	Cell Signaling	#Cat 8457S, RRID:AB_10950489
Streptavidin eFluor® 450	eBioscience	#Cat 48-4317-82, RRID:AB_10359737
VEGF-R2	Cell Signaling	#Cat 2479, RRID:AB_2212507
**Chemicals, peptides, and recombinant proteins**		
40/70 Percoll	GE Healthcare Life Sciences	#Cat 17-0891-01
7-Amino-Actinomycin D (7AAD)	Sigma-Aldrich	#Cat A9400
Accutase Cell Detachment Solution	Sigma-Aldrich	#Cat A6964
Albumin bovine (BSA) Fraction V	Biomol	#Cat 01400.100
Antigen unmasking Solution	Vector Laboratories	#Cat H-3300, RRID:AB_2336226
APC Calibrite beads	BD Biosciences	#Cat 340487, RRID:AB_2868735
Avidin/Biotin Blocking Kit	Vector Laboratories	#Cat SP-2001, RRID:AB_2336231

Cell Proliferation ELISA, BrdU (colorimetric)	Roche	#Cat 11647229001
Chloroform p.A.	Carl Roth	#Cat 3313.1
Collagenase Typ 4 CLS-4	Worthington	#Cat LS004189
Count bright absolute counting beads	Thermo Fisher	#Cat C36950
**Critical commercial assays**		
Antibody Diluent/Block	Perkin Elmer	#Cat ARD1001EA
AR6 Buffer	Perkin Elmer	#Cat AR600125ML
CXCR3 Inhibitor/(±)-AMG 487	Tocris Bioscience	#Cat 4487/10
Diethylnitrosamine	Sigma-Aldrich	#Cat N0756
DMEM, w: 4.5 g/L Glucose, w: L-Glutamine, w/o: Sodium pyruvate, w: 3.7 g/L NaHCO3	PAN BIOTECH	#Cat P04-03550
DMSO	Sigma-Aldrich	#Cat D2438
DNase1	Roche	#Cat 10104159001
Dulbecco´s Phosphate Buffered Saline (PBS), w/o: Ca and Mg	PAN BIOTECH	#Cat P04-36500
EDTA solution pH 8,0	PanReac AppliChem	#Cat A4892
Fixable Viability Dye eFluor450	eBioscience	#Cat 65-0863-18
Formaldehyde solution 4 % buffered	Otto Fischar GmbH & Co. KG	#Cat 27280
FoxP3 Trans. Staining Buffer	eBioscience	#Cat 00-5523-00
GoTaq qPCR Master Mix	Promega	#Cat A6002
Hemacolor quick stain kit (1-3)	Merck	#Cat 1.119.562.500<br>#Cat 1.119.572.500<br>#Cat 1119552500
Hepatotoxic carbon tetrachloride	Sigma-Aldrich	#Cat 289116
Hydrogen chloride (HCL) 1M	PanReac AppliChem	#Cat 1.810.211.211
Hydrogen peroxide 30% p.A.	Merck	#Cat 107209
Magnesium chloride hexahydrate	Carl Roth	#Cat 2189.2
Mowiol 4-88	Carl Roth	#Cat 0713.2
NaCl	Carl Roth	#Cat HN00.2
Natriumazid	Carl Roth	#Cat K305.1
Normal mouse serum	Abcam	#Cat ab7486
Penicillin-streptomycin	Life Technologies	#Cat 15140-122
Peroxidase Substrate Kit DAB	Vector Laboratories	#Cat SK-4100, RRID:AB_2336382
Pierce BCA Protein Assay Kit	Thermo Fisher	#Cat 23227
Prigrow X.1 Medium	abm	# Cat TM010

Proteinase K	Carl Roth	#Cat 7528.1
RBC lysis buffer (10x)	BioLegend	#Cat 420301
Recombinant murine IP-10/CXCL10	PeproTech	#Cat 250-16
Recombinant murine MIG/CXCL9	PeproTech	#Cat 250-18
Roti®-Histokitt (mounting medium for histology)	Carl Roth	#Cat 6638.1
RPMI 1640, w: L-Glutamine, w: 2.0 g/L NaHCO3	PAN BIOTECH	#Cat P04-16500
Sera Plus, EU approved regions, special processed FBS, 0.2 µm sterile filtered	PAN BIOTECH	#Cat P30-3702
Sirius Red F3B	Sigma-Aldrich	#Cat 365548
Sodium Hydroxide solution (1 M) for molecular biology	PanReac AppliChem	#Cat A6579
Tissue-Tek O.C.T Compound	Sakura	#Cat 4583
TRIS Pufferan 99,9%	Carl Roth	#Cat 4855.1
Triton X	Merck	#Cat 108603
TSA Cyanine 3	Perkin Elmer/ Akoya Bioscience	#Cat NEL704A001KT RRID:AB_2572409
TSA Cyanine 3.5	Perkin Elmer/ Akoya Bioscience	#Cat NEL763001KT
TSA Cyanine 5	Perkin Elmer/ Akoya Bioscience	#Cat NEL705A001KT
TSA Cyanine 5.5	Perkin Elmer/ Akoya Bioscience	#Cat NEL766001KT
TSA Fluorescein	Perkin Elmer/ Akoya Bioscience	#Cat NEL701A001KT
Tween 20	Carl Roth	#Cat 9127.1
Vecta shield Mounting Medium with DAPI	Vector Laboratories	#Cat H-1200, RRID:AB_2336790
Xylol	Fischar	#Cat 27404
**Experimental models: Cell lines**		
Human HUH7 cells / well differentiated hepatocyte-derived carcinoma cell line		P+4, RRID:CVCL_0327
HUVEC / human umbilical vein endothelial cells		P+X, RRID:CVCL_LF80

Mouse: *Cxcl10*$^{-/-}$ and WT (C57Bl/6J) littermates	University hospital Aachen, Animal facility	[15]
**Oligonucleotides**		
18S<br>CTCAACACGGGAAACCTCAC (for)<br>CGCTCCACCAACTAAGAACG (rev)	N/A	Eurofins MWG Operon
Afp<br>AGCAAAGCTGCGCTCTCTAC (for)<br>AGGGGCTTTCCTCGTGTAAC (rev)	N/A	Eurofins MWG Operon
Ctla-4<br>TTTTGTAGCCCTGCTCACTCT (for)<br>CTGAAGGTTGGGTCACCTGTA (rev)	N/A	Eurofins MWG Operon
Cxcl10<br>CCAAGTGCTGCCGTCATTTTC (for)<br>GGCTCGCAGGGATGATTTCAA (rev)	N/A	Eurofins MWG Operon
Cxcl9<br>TAGGCAGGTTTGATCTCCGT (for)<br>CGATCCACTACAAATCCCTCA (rev)	N/A	Eurofins MWG Operon
Cxcr3<br>AGAATCATCCTGGTCTGAGACAA (for)<br>AAGATAGGGCATGGCAGCTA (rev)	N/A	Eurofins MWG Operon
Hgf<br>GATGAGTGTGCCAACAGGTG (for)<br>GGTCAAATTCATGGCCAAAC (rev)	N/A	Eurofins MWG Operon
Il10<br>GCCAAGCCTTATCGGAAATGA (for)<br>CTTGATTTCTGGGCCATGCT (rev)	N/A	Eurofins MWG Operon
Il12<br>CTCTGAATCATAATGGCGAGACT (for)<br>TTCACTCTGTAAGGGTCTGCTTC (rev)	N/A	Eurofins MWG Operon
Il13<br>TGGATTCCCTGACCAACATCTC (for)<br>GGTTACAGAGGCCATGCAATATC (rev)	N/A	Eurofins MWG Operon
Il4<br>AAACTCCATGCTTGAAGAAGAACT (for)<br>CTACGAGTAATCCATTTGCATGAT (rev)	N/A	Eurofins MWG Operon
TTAGATGTGGAGCAACTTGGAAT (for)<br>CATTCTTTGCACAGGATATCACA (rev)	N/A	Eurofins MWG Operon
Pd-l1<br>GCTCCAAAGGACTTGTACGTG (for)<br>TGATCTGAAGGGCAGCATTTC (rev)	N/A	Eurofins MWG Operon
GAGCCCAATCACGTTTCAGTTT (for)<br>TCCTTCCTGCTTCTTGCTAGCT (rev)	N/A	Eurofins MWG Operon

Tgfß<br>GAAGGACCTGGGTTGGAAGT (for)<br>GTTGGTTGTAGAGGGCAAGG (rev)	N/A	Eurofins MWG Operon
CGCGAGGTTGTTTAGAGTAGCA (for)<br>TCAGTCCAAGCAACACTCTCTGAT (rev)	N/A	Eurofins MWG Operon
CGGAAGATTAGGGTTGTTTCTG (for)<br>TCCACAGCAGTCAAACATATCC (rev)	N/A	Eurofins MWG Operon
Vegf-r2<br>GCCCTGCTGTGGTCTCACTAC (for)<br>CAAAGCATTGCCCATTCGAT (rev)	N/A	Eurofins MWG Operon
**Software and algorithms**		
FlowJo software v10.6.0	FLOWJO LLC	https://www.flowjo.com/solutions/flowjo
Fiji - Image J	Schindelin *et al.,* 2012 [21]	https://imagej.net/Fiji/Downloads
Kaplan-Meier Plotter online tool	Menyhart O *et al.* R Soc Open Sci 2018;5:181006 .	This paper
GraphPad Prism 9.0	GraphPad	https://www.graphpad.com/scientific-software/prism/
BD FACSDiva V10 software	BD Bioscience	https://www.bdbiosciences.com/en-au/products/software/flowjo-v10-software
Vectra 3.0 Automated Quantitative Pathology Imaging System	PerkinElmer	
R	RStudio	https://www.rstudio.com/

A

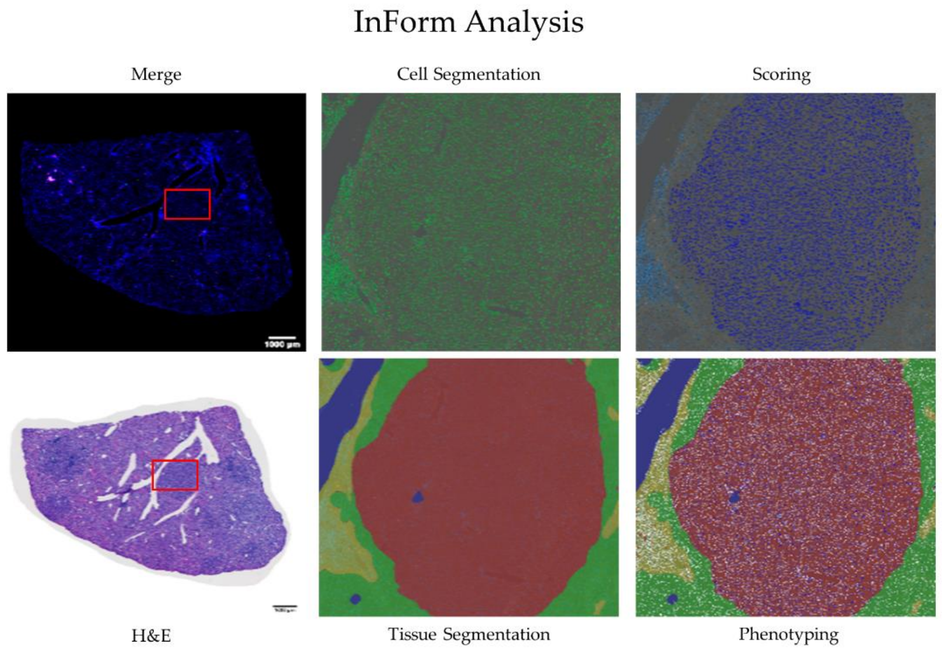

B

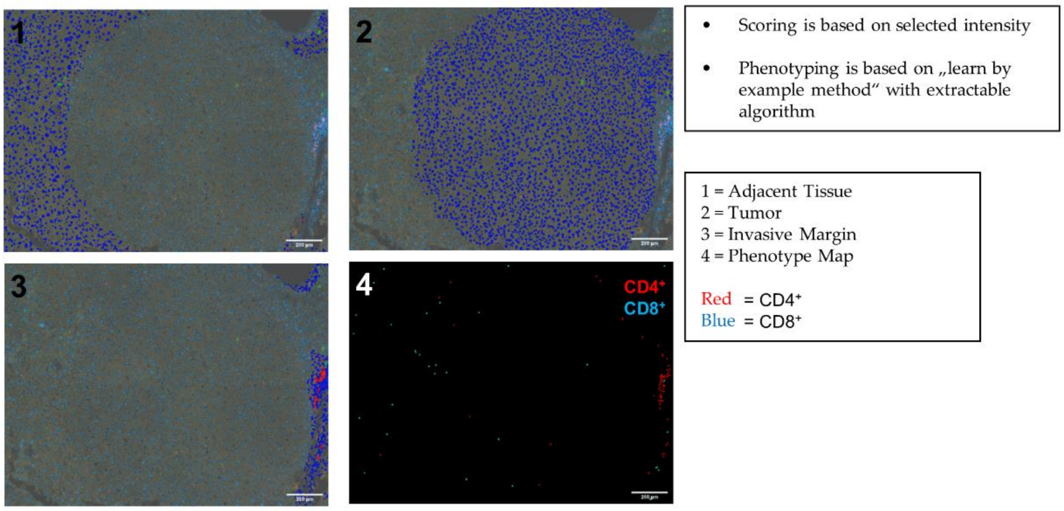

Figure S1. Multiplex staining InForm Analysis, Phenotyping and Scoring (A) InForm analysis of Multiplex staining. Representative multiplex staining, H&E staining, cell and tissue segmentation image as well as scoring and and phenotyping of tumorigenic liver tissue, scale bar 1000 µm (B) Exemplary scoring of CD4+ and cleaved Caspase 3+ cells from the cleaved Caspase 3 multiplex staining for inForm analysis. Positivity threshold was manually set for cells to be detected as CD4 (red) or cleaved Caspase 3 (green) positive. Scoring was done for the different tissue regions: (1) adjacent tissue, (2) tumor tissue and (3) invasive margin. scale bar 200 µm

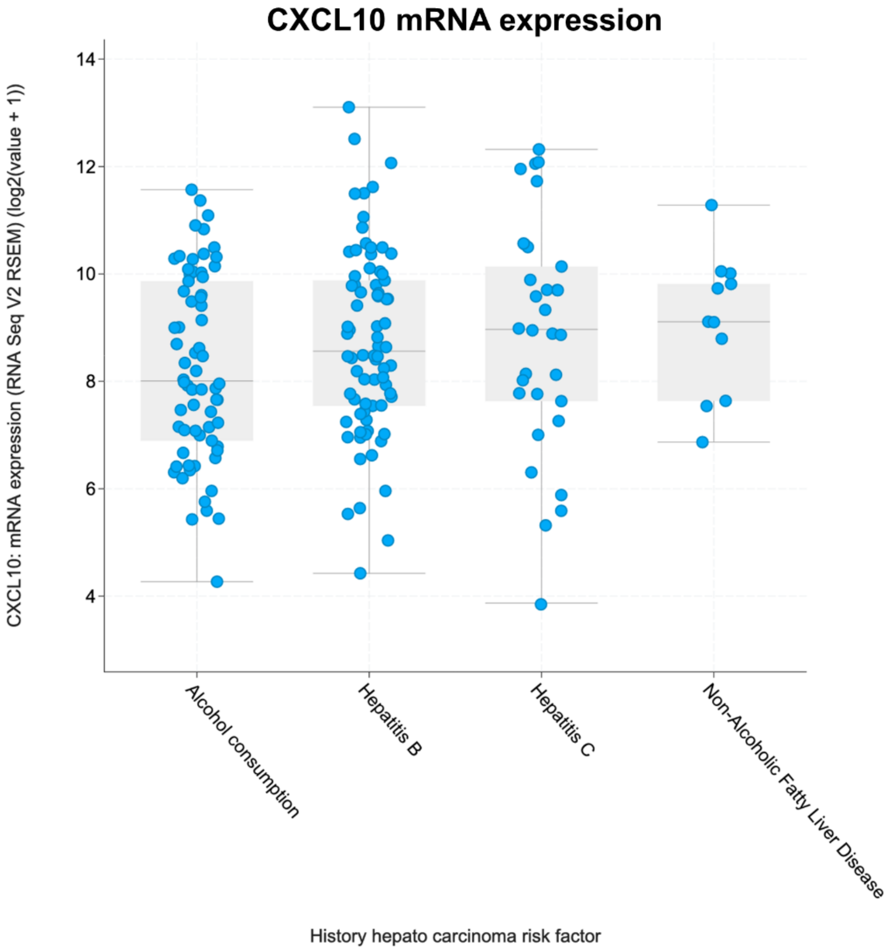

Figure S2. Intratumoral Cxcl10 mRNA expression stratified depending on HCC risk factor in a cohort of 371 HCC patients (TCGA firehose legacy datasets), plot is generated using the open-access cBioPortal (Cerami et al. The cBio Cancer Genomics Portal: An Open Platform for Exploring Multidimensional Cancer Genomics Data. Cancer Discovery. May 2012 2; 401.; Gao et al. Integrative analysis of complex cancer genomics and clinical profiles using the cBioPortal. Sci. Signal. 6, pl1 2013)

.

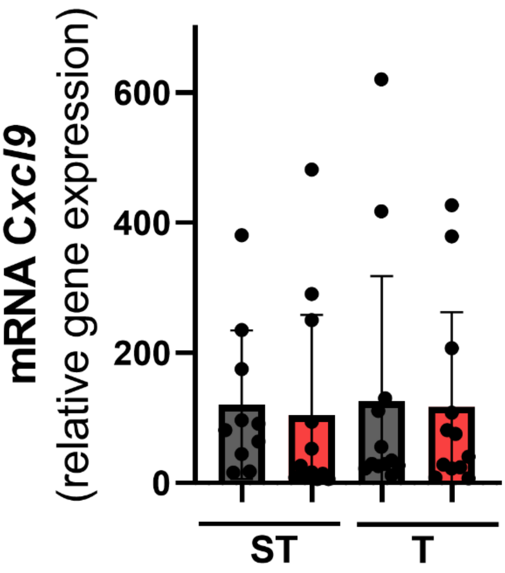

Figure S3. mRNA expression level Cxcl9 was quantified by qRT-PCR of non-tumor liver surrounding tissue (ST) as well as tumor tissue (T) of WT and *Cxcl10*$^{-/-}$ mice. The amount of mRNA expression was normalized to the expression of *18S*. Data are expressed as mean ± SEM.
